# Distinguishing Social From Private Intentions Through the Passive Observation of Gaze Cues

**DOI:** 10.3389/fnhum.2019.00442

**Published:** 2019-12-17

**Authors:** Mathis Jording, Denis Engemann, Hannah Eckert, Gary Bente, Kai Vogeley

**Affiliations:** ^1^Cognitive Neuroscience (INM-3), Institute of Neuroscience and Medicine, Research Center Jülich, Jülich, Germany; ^2^Department of Psychiatry and Psychotherapy, University Hospital Cologne, Cologne, Germany; ^3^Université Paris-Saclay, Inria, CEA, Palaiseau, France; ^4^Department of Communication, Michigan State University, East Lansing, MI, United States

**Keywords:** social gaze, Bayesian multilevel models, ostension, eye contact, communicative intention, gaze cueing

## Abstract

Observing others’ gaze is most informative during social encounters between humans: We can learn about potentially salient objects in the shared environment, infer others’ mental states and detect their communicative intentions. We almost automatically follow the gaze of others in order to check the relevance of the target of the other’s attention. This phenomenon called gaze cueing can be conceptualized as a triadic interaction involving a gaze initiator, a gaze follower and a gaze target, i.e., an object or person of interest in the environment. Gaze cueing can occur as “gaze pointing” with a communicative or “social” intention by the initiator, telling the observer that she/he is meant to follow, or as an incidental event, in which the observer follows spontaneously without any intention of the observed person. Here, we investigate which gaze cues let an observer ascribe a social intention to the observed person’s gaze and whether and to which degree previous eye contact in combination with an object fixation contributes to this ascription. We varied the orientation of the starting position of gaze toward the observer and the orientation of the end position of a lateral gaze shift. In two experiments participants had to infer from the gaze behavior either mere approach (“the person looked at me”) vs. a social (“the person wanted to show me something”) or a social vs. a private motivation (“the person was interested in something”). Participants differentially attributed either approach behavior, a social, or a private intention to the agent solely based on the passive observation of the two specific gaze cues of start and end position. While for the attribution of privately motivated behavior, participants relied solely on the end position of the gaze shift, the social interpretation of the observed behavior depended additionally upon initial eye contact. Implications of these results for future social gaze and social cognition research in general are discussed.

## Introduction

The eye region displays emotional and attentional states and is a crucial element in understanding the inner experiences of others (Baron-Cohen et al., 1997; Emery, 2000). This leads to the pivotal role of gaze in social cognition research (Shepherd, 2010) because it informs not only about internal states of persons but also about their relationship to objects or persons in their environment. Humans process the gaze direction, deduce from it the focus of attention and automatically shift their own attention accordingly. This process is called gaze cueing (Frischen et al., 2007) and is a prerequisite for joint attention, the case in which both persons visually attend the same object. Observing someone looking at objects also informs us about the environment shared by both partners. Accordingly, following someone’s gaze changes the perception and processing of jointly attended objects (Becchio et al., 2008); objects, that had previously been looked at by another person are liked more (Bayliss et al., 2006). Gaze following is acquired early in life: 6 month old infants are already able to follow someone’s gaze (Senju and Csibra, 2008). Proficiency in gaze following predicts the development of language “theory of mind” capacity (Morales et al., 1998), IQ, self-regulation, social competence and depth of information processing (Mundy and Newell, 2007). It is also believed to be a prerequisite component for reinforcement learning (Vernetti et al., 2017).

A key research question is whether successful gaze processing is an automatic holistic ability, or whether it can be decomposed into distinct cognitive operations, hence, taught and learned. As a clear prerequisite, the gaze angle has to be estimated and the spatial location of the partner’s attention has to be inferred from the gaze vector. Compared to great apes and monkeys, humans are especially proficient in this regard (Gibson and Pick, 1963), and the neural implementation of gaze reconstruction has been intensely researched over the past decades (Itier and Batty, 2009).

A second challenge is to discern intentions underlying gaze behavior, which may be explicitly communicative or “social” in the sense that gaze partners want to convey certain information. The “dual function” of gaze comprises the perception of the environment and the signaling of the attentional focus to others (Gobel et al., 2015). I.e., we do not only use the gaze of others as a cue about their attentional focus, but we are at the same time aware that others can deduce our attentional focus from our gaze. Effects of this awareness have been demonstrated impressively in studies showing that participants control their gaze according to its social adequacy when being watched (Risko and Kingstone, 2011). In other words, humans are forced to actively avoid undesired communication by controlling their eye gaze in social contexts. Likewise, when observing another person, this person’s gaze might be driven by self-centered interests or it might be an attempt to communicate or to express a “social” intention. Thus when deducing the other’s intentions, perceivers have to distinguish between “private” and “communicative” intentions (Walter et al., 2004). It can be expected that this distinction fundamentally affects our relationship toward the other person. Walter et al. (2004) could show that, during mentalizing, the processing of private and communicative intentions rely on distinct neural mechanisms, even if the communicative actions are not directed toward the observer.

Csibra and Gergely (2009, 2011) speculate that humans use eye contact as an “ostensive” signal to announce situations in which they want to show or teach something to others. Being gazed at by another person is a powerful social cue to which most humans are highly sensitive (von Griinau and Anston, 1995; Senju and Johnson, 2009), and eye contact is supposed to signal communicative intents (Kleinke, 1986). Conversely, according to Csibra and Gergely (2009, 2011), infants have an innate sensibility to ostensive cues which allows them to generalize their experience in these situations in order to fully benefit from their teacher. Preceding communication indeed has been shown to facilitate subsequent gaze cueing and gaze following already in 4–6 month old infants (Farroni et al., 2003). This mechanism might also explain the strong ontogenetic link not only between gaze following and joint attention, but also between the mental and cognitive development.

Here, we present two studies that explore the link between gaze direction processing and communicative or “social” affordances. We investigate the principles of how humans deduce the attentional focus from others’ gaze with regard to the tension between private and communicative or “social” intentions. The motivation for Study 1 was to study the role of eye contact in reducing the ambiguity of gaze and to identify the parameters that allow to interpret the gaze behavior of others as ostensive, i.e., a special case of communicative intention that bridges the gap between person and environment. Specifically, we aimed at the difference between situations in which we experience an interacting partner as being interested in us by visually attending to us in contrast to situations in which the partner is actively trying to communicate with us about something in the outside world by a rudimentary form of joint attention. The observation of distinct patterns of observed gaze in the two conditions lead us to the question in Study 2, whether and how participants distinguish aforementioned communicative intentions from situations in which the partner is experienced as being “privately” interested in something without involving and addressing the perceiver.

**FIGURE 1:**
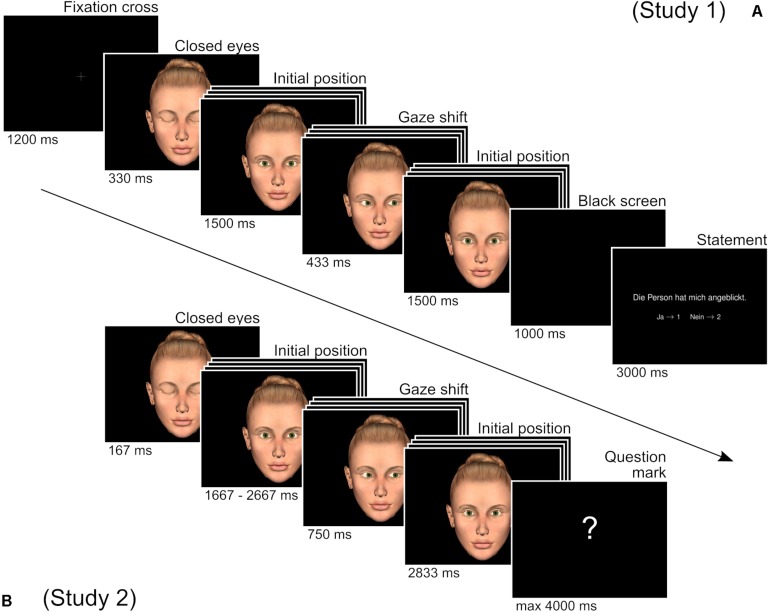
Course of one trial of Study 1 (A) and Study 2 (B). The stack of images for initial position and shift position indicate that in each trial, one out of four possible images was displayed. Note that after the shift, the VC always returned to the same initial position it had started from. In this example, the initial position 1 (direct gaze) and the shift position 4 are depicted. The question mark indicated the prompt for participants to give their ratings.

As the basic design of both studies, participants watched short videos of a virtual character (VC) looking at the participant with different degrees of vertical deviations, ranging from direct gaze (i.e., eye contact) to different degrees of downward averted gaze, before shifting the gaze to the left or to the right with different degrees of lateral deviations. (For simplicity, we will refer to the starting position of initial gaze as “initial position” and to the gaze shift to the left or to the right as “shift amplitude”). Subsequently, participants had to report their experiences based on explicit statements (see Figure 1). We used VCs as stimulus material, as they combine high experimental control with ecological validity (Vogeley and Bente, 2010) and are well suited for the investigation of non-verbal communication (Pfeiffer et al., 2013, 2014; Georgescu et al., 2014; Jording et al., 2019).

In the first study, we investigated the difference between situations in which participants had the impression of been looked at by the VC (“LOOK” condition) and situations in which they had the impression that the VC was trying to show them something (“COM,” e.g., “communicative,” condition). Besides the aforementioned empirical question, a second goal of this first study was to ensure the validity of our stimuli and the overall methods. The sensitivity of human observers to the visual stimulus of eyes directed at them is already well established (Senju and Johnson, 2009) and VCs were shown to reliably induce the impression of social presence (Bente et al., 2007). Our stimuli can elicit the feeling of being in the attentional focus of or being addressed by the VC. Therefore, results of this first study serve as a test of these properties of the stimuli. This first study was conducted as an internet-based online survey to maximize sample size and account for possible variability in the general population. In the second study with a new sample of participants, we again studied COM in comparison to the situation in which the VC was merely privately interested in something without any social intention (“PRIV” condition). This second study had a repeated measures design and was conducted in a laboratory setting, increasing experimental control of environment and participant specific factors.

We expected the impression of being looked at to be dependent solely on the degree to which the initial gaze is directed toward the participants but not on subsequent outward-directed behavior. In COM, available evidence in the field suggested an influence of preceding eye contact for the impression of communicative intentions as well. Considering that participants were asked whether the other wanted to show them something located in the outside world, we also expected an influence of the subsequent gaze shift during COM. However, this situation by definition requires a triadic interaction between two interactants and another object in the environment. Therefore, we expected high agreement rates only for situations with direct gaze and large shift amplitudes. During PRIV, we expected an influence of the shift amplitude only. However, it was also interesting to see whether preceding eye contact might have an adverse effect. Should participants understand private and communicative intentions as mutually exclusive, they should take eye contact as an indicator of the latter, leading to an impediment of the impression of mere personal interest.

## Materials and Methods

### Study 1

#### Participants for Study 1

Out of 555 participants, 403 participants completed the online survey. In 11 cases videos were not presented correctly, resulting in 392 remaining participants (257 female; age ranging from 17–70 years, *M* = 30, SD = 10.63). Participants were recruited via mailing lists from different German universities (University of Cologne, University of Münster, University of Bayreuth) and gave their informed consent prior to participation. There were no further exclusion criteria.

#### Stimuli for Study 1

One female and one male VC were created with Poser for Apple Mac OS X (Poser 8, Smith Micro Software, Inc., Columbia, SA, United States). For both VCs images were created for four different initial gaze positions and for four different gaze shift targets in two different directions. Initial gaze positions were equidistantly positioned on a central vertical line, ranging from direct gaze to clearly averted gaze. Positions after gaze shifts were equidistantly located on a horizontal line slightly below the eye level, ranging from slight central deviation up to the maximal still realistic and lifelike appearing deviation, both for the right and the left side. From these images we approximated the deviation of the visual angle from direct gaze (initial position 1) by measuring for all images the position of the iris in relation to its position in the direct gaze image. On this basis we computed angles, taking 22 mm as the average diameter of the human eye (Bekerman et al., 2014) and 12 mm as average diameter of the human iris (Thainimit et al., 2013). Averaged between VCs, the initial positions vertically deviated approximately equidistantly from direct gaze by 0°, 3°, 8°, and 12°. VC-averaged gaze positions after the shift lay on a plane 6° vertically below the eye level, horizontally deviating from direct gaze approximately equidistantly by 5°, 9°, 14°, and 18°. (For examples of all initial positions and gaze shift images and the exact values of the degree of aversion, please refer to the Supplementary Material.) Images of initial positions and gaze shifts were then combined to flash videos by the python 2.6 based video tool “ffmpeg 0.7.8.” For both sexes of VCs videos were created for each combination of four different initial positions and four different shift amplitudes to both sides, resulting in 16 videos of gaze shifts to the right and 16 videos for gaze shifts to the left per VC and a total of 64 videos. Each video started with showing a fixation cross for 1200 ms. Afterward the VC appeared, having his/her eyes closed for 330 ms before he/she subsequently opened the eyes and looked toward the initial position for 1500 ms, then shifted toward the target for 433 ms, before returning to the initial position for 2000 ms. Afterward the screen went black for 1000 ms, before the statement and response buttons were displayed for 3000 ms as a reminder at the end of the video (see Figure 1A for an illustration and Supplementary Videos S1–S4 for examples of the trial course).

#### Task for Study 1

Each participant watched videos of either the female or the male VC for all 16 different combinations of gaze initial positions and shift amplitudes to the left or to the right in randomized order exactly once. After each video participants had to rate the VCs behavior according to statements randomly assigned in the beginning of the experiment. Statements were either “the person looked at me” (German original: “Die Person hat mich angeblickt”) or “the person wanted to show me something” (German: “Die Person wollte mir etwas zeigen”), to which participant had to respond per button press in a binary choice (“yes” or “no”).

#### Setup and Design for Study 1

The survey was presented via the online survey tool Unipark (Questback GmbH, Cologne, Germany). During the survey, participants were informed about the procedure, the voluntary nature of their participation and the opportunity to withdraw from the study at any point in time and without providing any reasons for their decision. They further had to state their age and sex before they were pseudo-randomly assigned to one of the two VCs and one of the two rating statements. After that, participants were told which statement they had to answer and whether they would see a female or a male character. Participants were then presented with videos for all 16 combinations of initial positions and shift amplitudes in a pseudorandomized order with shifts randomly either to the right or the left. After each video the screen turned black before the statement was presented together with the binary response options (button “1” for “Yes” and button “2” for “No”). The next trial then started after the participants had given their answers.

#### Statistics for Study 1

The effect of different gaze shifts (initial position and shift amplitude) on the ascription of different intentions to the VC (conditions) were analyzed in a multilevel model with an inverse logit link function, in which we considered individual differences between the participants’ average responses through varying intercept coefficients. Importantly, we considered the statement as experimental condition and hence constructed a joint model for both statements instead of two separate models. The model focuses on the interaction between the statement and eye gaze behavior. This approach has enabled explicitly modeling statement-specific-biases, e.g., due to difficulty or individual preferences, while, at the same time subjecting the estimated differences between the effects to statistical control through shrinkage priors (see below). The resulting logistic regression model can be expressed as:

$$y_{i}∼Binomial\left(n=1,p={\hat{y}}_{i}\right)$$

$${\hat{y}}_{i}=l\,o\,g\,i\,t^{-1}\,\left(\alpha_{j}\,\left[i\right]+T\,\left[i\right]*\beta \right)$$

Where α_*j*_ is the individual intercept for each subject, *T* is a matrix of treatment effects, and β the unknown parameter vector that has to be learnt from the data. The treatment effects are the statement, the vertical initial gaze position and the horizontal amplitude of the gaze shift, covering all main effects as well as second and third order interactions. The statement was dummy-coded with a 0–1 predictor. We included the eye gaze as continuous predictor after z-scoring. No prior information concerning effect sizes of the initial gaze position or shift amplitude were available. We hence used the non-informative default priors from the “brms” package (Bürkner, 2017, 2018) according to which coefficient are centered around zero. These priors are shrinkage priors and are conservative. Shrinkage is used in statistics to improve generalization to new data can be thought of correcting initial estimates by pushing them toward zero. The amount of shrinkage fades out as the sample size increases. For the prior for the population variance component σ_*j*_ of the individual intercepts, we kept the conservative default prior that puts most probability mass on smaller values close to zero.

$$\beta ∼student^{\prime }st\left(df=3,center=0,\sigma^{2}=10\right)$$

$$\alpha_{j}∼student^{\prime }st\left(df=3,center=0,\sigma^{2}=\sigma_{j}\right)$$

$$\sigma_{j}∼half\text{-}student^{\prime }st\left(df=3,center=0,\sigma^{2}=\sigma_{j}\right)$$

Note that the population variance parameter σ_*j*_ uses the upper half of the student-t distribution due to the constraint that the variance cannot be negative. Also note that σ_*j*_ is a hyper-parameter and has to be estimated from the data. Here, it controls how much the model trusts the individual intercept estimates σ_*j*_ and to which extent these will be corrected by shrinkage toward the global intercept. Smaller values for σ_*j*_ would produce stronger shrinkage. This is a core feature of the multilevel model and is also referred to as partial pooling (Gelman, 2006).

We performed prior predictive checks to ensure that the priors are approximately uninformative on the scale of the model predictions after the inverse logistic link function. Analysis revealed that the results were insensitive to the choice of the prior due to the size of the data set. Data were analyzed using the “rstan” (Stan Development Team, 2018) and “brms” (Bürkner, 2017, 2018) packages for the programing language R for statistical computing (R Development Core Team, 2008) and RStudio (R Studio Team, 2016). Model fitting was performed using a Hamilton Markov chain Monte Carlo algorithm (Hoffman and Gelman, 2014). Models were run with 1000 warmup samples and 1000 iterations in total, using four chains, yielding 4000 draws from the approximated posterior distribution. Successful convergence was assessed based on the potential scale reduction factor $\hat{R}$, also known as the Gelman-Rubin statistic. $\hat{R}$, was found to be acceptably close to 1.0 (±0.1) for every model (see Supplementary Table S1). Posterior distributions were visually compared to observed data in order to check consistency.

### Study 2

#### Participants for Study 2

34 subjects (19 female; age range 21–54, *M* = 28.88, SD = 5.82; not out of the sample from Study 1) participated in this experiment. None of these participants met any of the exclusion criteria (depressive symptoms as indicated by BDI scores: *M* = 3.79, range = 0–17, cut-off ≥ 19; autistic traits as indicated by AQ scores: *M* = 10.42, range = 2–19, cut-off ≥ 32; general cognitive impairments as indicated by MWT: *M* = 112.59, range = 97–136, cut-off < 70, or KAI, *M* = 124,24, range = 100–143, cut-off < 70) so that all participants were included for further analysis. The mean empathy score of the resulting sample as indicated by the SPF was *M* = 40.64, range = 30–49. Participants were recruited via mailing lists from the University of Cologne and gave their informed consent before participating.

#### Stimuli for Study 2

The same VC pictures were used as in Study 1. Instead of beforehand creating animated videos, as in Study 1, images were now combined to animations within the presentation software (Python 2.6), allowing for jittering of presentation durations. As in Study 1, animations of both VCs could be presented displaying gaze shifts for all 16 possible combinations of initial positions and shift amplitudes to both directions (left and right), resulting in a total of 32 different gaze shifts per VC. Each video sequence started with the VC having its eyes closed for 167 ms (10 frames) before opening them and looking toward the gaze initial position for 1667–2667 ms (100–160 frames). Afterward the VC gaze shifted and then stayed at the new location for 750 ms (45 frames) before returning to the initial location at the end of the video for another 2833 ms (170 frames). Subsequently, a screen showing a white question mark in front of a black background requested the participants to give their answer for a maximum of 4000 ms. (Please refer to Figure 1B for an illustration and Supplementary Videos 5–8 for examples of the trial course).

#### Task for Study 2

In accordance with Study 1, participants, after having watched a gaze shift performed by the VC, had to rate the VCs behavior according to one of two different statements per trial. The statements were either “the person wanted to show me something” (German: “Die Person wollte mir etwas zeigen”) or “the person was interested in something” (German original: “Die Person interessierte sich für etwas”). Again, participants had to respond per button press in a binary choice (“Yes” or “No”), for which they had 4 s before the next trial would start.

#### Setup and Design for Study 2

Before the experiment started, participants general cognitive level was assessed by two tests: KAI (Lehrl et al., 1991) and MWT-B (Lehrl, 2005). The experiment was conducted on a Lenovo ThinkPad T410 (Intel Core i5-520 M, 2,4 Ghz, 4GB RAM; OS: Ubuntu Linux 12.4 LTS) and displayed on a Tobii T60 Eye Tracker (60 Hz refresh rate, 1280 × 1024 px resolution) with responses given via keypad buttons and instructions presented on the screen. For the experiment, two blocks of trials (one block per statement) were presented in a pseudorandomized fashion. In each block, the participant watched all 64 gaze shifts (four initial positions × four shift amplitudes × two directions × two VCs) resulting in a total of 128 trials per participant over the whole experiment and a total duration of approximately 20 min. Before the experiment, KAI (Lehrl et al., 1991) and MWT-B (Lehrl, 2005) were conducted to rule out general cognitive impairments. After the experiment participants completed BDI (Beck et al., 2001), and AQ (Baron-Cohen et al., 2001) to rule out depressive and autism-like syndromes, respectively. In addition, participants filled out the empathy inventory SPF (Paulus, 2009) to potentially allow the matching with patient samples in future clinical studies.

#### Statistics for Study 2

The same statistical procedures where applied as in Study 1 (for $\hat{R}$ values see Supplementary Table S2). Note that the multilevel approach has allowed us to use the same model specification for Study 2, as this kind of model is robust to the structure of repeated observations and can be applied to a wide array of between or within-subject designs (see McElreath, 2016, Chapter 12, box on pp. 371 for discussion).

## Results

**FIGURE 2:**
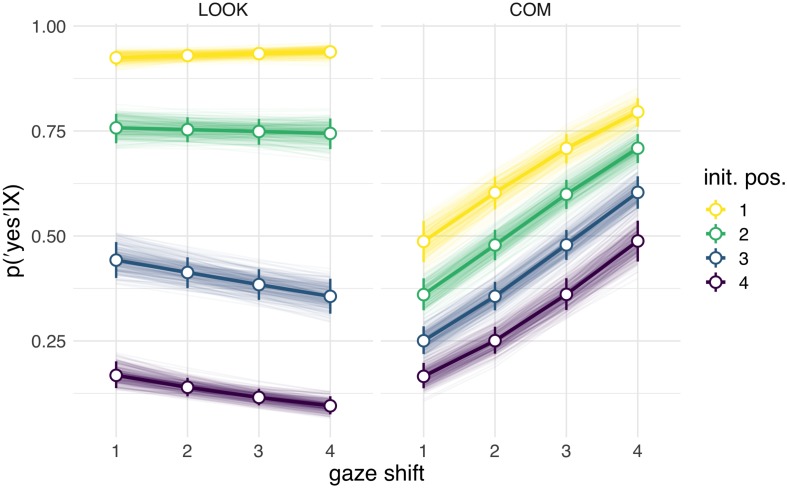
Posterior predictions of the influence of initial position (,,init. pos.“) and gaze shift amplitude (,,gaze shift“) in Study 1 in the LOOK condition (“the person looked at me”) and the COM condition (“the person wanted to show me something”). For the initial position, „1“ corresponds to direct gaze and „4“ to a maximally (vertically) averted position. For the shift amplitude, „1“ corresponds to the smallest and „4“ to the largest possible shifts.

**FIGURE 3:**
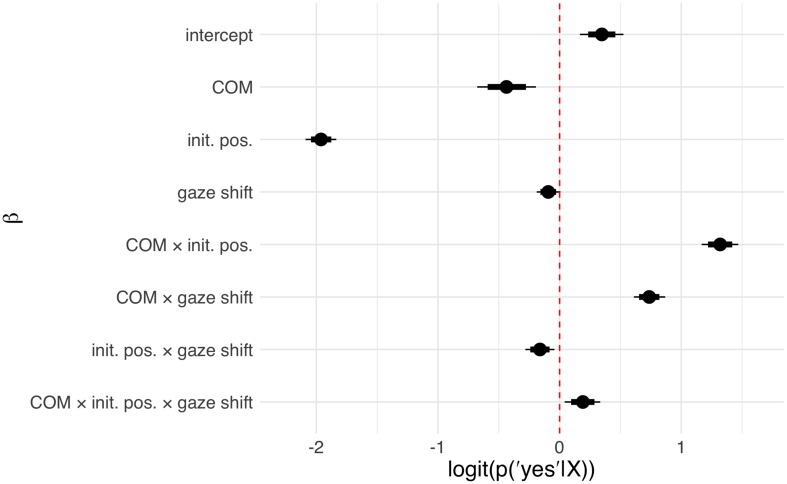
Coefficients sampled from the approximate posterior distribution in Study 1 for the influence of condition, initial position, shift amplitude, and their respective interactions. Circles depict the posterior mean, horizontal bars and lines denote the 80 and 95% posterior compatibility intervals, respectively. The COM coefficient describes the effect of the COM condition in contrast to the LOOK condition. The coefficient for initial positions depicts the stepwise effect of increasing aversion from direct gaze in the initial position (farther from direct gaze). The coefficient of shift amplitude depicts the stepwise effect of increasing the shift amplitude. For additional statistics see Supplementary Table S1; Note that although not apparent here, the 95% confidence interval of the gaze shift coefficient does include zero.

**FIGURE 4:**
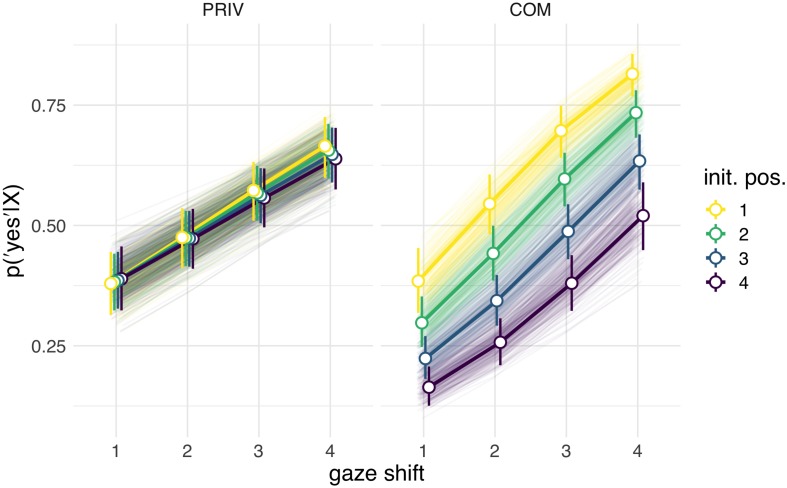
Posterior predictions of the influence of initial position („init. pos.“) and gaze shift amplitude („gaze shift“) in Study 2 in the PRIV condition (“the person was interested in something”) and the COM condition (“the person wanted to show me something”). For the initial position, „1“ corresponds to direct gaze and „4“ to a maximally (vertically) averted position. For the shift amplitude, „1“ corresponds to the smallest and „4“ to the largest possible shifts.

**FIGURE 5:**
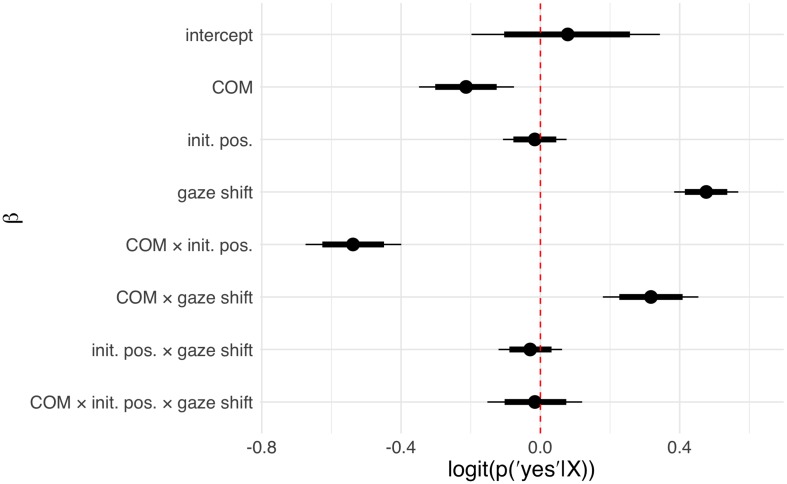
Coefficients sampled from the approximate posterior distribution in Study 2 for the influence of condition, initial position, shift amplitude, and their respective interactions. Circles depict the posterior mean, horizontal bars and lines denote the 80 and 95% posterior compatibility intervals, respectively. The COM coefficient describes the effect of the COM condition in contrast to the PRIV condition. The coefficient for initial positions depicts the stepwise effect of increasing aversion from direct gaze in the initial position (farther from direct gaze). The coefficient of shift amplitude depicts the stepwise effect of increasing the shift amplitude. For additional statistics see Supplementary Table S2.

Interpreting multilevel models solely based on their coefficients is known to be notoriously difficult, especially for generalized linear models with non-Gaussian probability models (Ai and Norton, 2003). As is common practice, we therefore considered posterior predictions (Figure 2 for Study 1; Figure 4 for Study 2) in addition to model coefficients (Figure 3, Study 1; Figure 5, Study 2). The posterior predictions contain the uncertainty of the model and can be readily interpreted in terms of the probability of the responses given the model and the data. They conveniently support statistical inference and can be analyzed in terms of percentiles or subtracted from another to form contrasts. For the effect of the individual predictors, beta coefficients (as well as the respective 80 and 95% posterior probability distribution intervals) are reported in the Figures 3, 5, additional statistics can be found in the Supplementary Tables S1, S2. This approach was chosen in order to increase the comparability to traditional reports of frequentist statistical methods with 0.05 significance levels. The intercepts for Study 1 and 2 refer to the COM condition, coefficients for the LOOK condition (Study 1) or the PRIV condition (Study 2) describe the change in coefficients compared to this intercept.

### Study 1

In Study 1 (online study) 198 participants (134 female, 64 male; age: 17–66 years, *M* = 29.37, SD = 10.69) participated in the LOOK condition and 194 participants (123 female, 71 male; age: 18–70 years, *M* = 30.07, SD = 10.59) participated in the COM condition. We compared posterior predictions for agreements to LOOK (“the person looked at me”) and COM (“the person tried to show me something”) statements (Figure 2). Posterior predictions revealed that participants discriminated the two conditions based on the two gaze dimensions, initial position and shift amplitude (Figure 2). In the COM condition, the effect of initial position as well as shift amplitude had substantial effects with the probability of agreement to the statement “the person tried to show me something” increasing with initial positions closer to eye contact and larger shift amplitudes. In comparison, in the LOOK condition, the effect of the initial position was even more pronounced while the shift amplitude did not show any considerable effect on the probability of “the person looked at me” statement. In addition, a slight tendency to higher overall agreements to the LOOK compared to the COM statements is visible. These results are reflected in the configuration of the model coefficients (Figure 3) which uncovered higher order interaction effects between condition and the dimension of gaze shifts.

### Study 2

In Study 2 (Lab Study) all 34 subjects participated in both conditions (COM and PRIV) in a repeated measures design. Here, we tested whether results from the COM condition in Study 1 could be replicated and how they would compare to the PRIV condition. In posterior predictions (Figure 4) for the COM condition the same pattern as in Study 1 arose with the probability of agreeing with the statement “the person tried to show me something” increasing with initial positions closer to eye contact and with larger shift amplitudes. Corroborating results of Study 1, no considerable interaction effect between initial position and shift amplitude was observed. In comparison, posterior predictions for the PRIV condition revealed that the overall tendency to agree with the statement “the person was interested in something” was slightly higher. Larger shift amplitude enhanced the probability of agreement even further, although this effect was less pronounced in PRIV compared to COM. Neither the initial position nor the interaction between initial position and shift amplitude had considerable effects in PRIV. Results correspond to the configuration of model coefficients (Figure 5), which uncovered simple but no higher order interactions.

## Discussion

The present study focused on the interplay of person-related and environment-related aspects of gaze behavior and how they influence our tendency to ascribe communicative or “social” and “private” intentions. The impression of being looked at (LOOK) has proved to be highly relying on initial eye contact for only in the conditions of direct gaze (or only slightly diverted gaze) ratings reached at least 75% agreement rates, while in cases of more diversion, agreement decreased substantially. Given the high sensitivity of humans to eye contact (von Griinau and Anston, 1995; Senju and Johnson, 2009) and its close link to intimacy (Argyle and Dean, 1965) this finding appears highly plausible. The amplitude of the subsequent gaze shift had no decisive influence, which corresponded also with our expectation.

The communicative condition (COM) revealed substantially the same results in the online study as in the laboratory study. Here, direct gaze or starting points close to it during the initial gaze and large gaze shifts significantly fostered the impression of being shown something. This matches the role of eye contact conveying communicative intentions (Kleinke, 1986) and nicely fits accounts of eye contact being used as ostensive cue. However, the ostensive situation also extends beyond the dyadic interaction of the two persons to the outside world. This is represented in the increasing effect of the assumed goal-directedness of the gaze shift. In other words, gaze contact with the viewer is only one component, the other component that makes this gaze behavior ostensive, is obviously the gaze shift directed toward an invisibly target in the environment. This result also ties in with other findings showing that infants as young as 9 month are not only sensitive to ostensive gaze cues, but they also expect object directed gaze shifts in these situations (Senju et al., 2008). Similarly, we had expected that participants would experience communicative intentions only when the triadic nature of the situation was apparent in the agents’ gaze behavior. Accordingly, we expected to find an interaction effect between the degree of eye contact and shift amplitude for the COM condition. However, this interaction effect proved to be negligible compared to the observable main effects. Thus, in our initial hypothesis we overestimated the component to which participants considered contextual factors when inferring communicative intentions. The question therefore remains, to which extent the effect of ostensive signals facilitating gaze cueing can be ascribed to more fundamental levels of processing. When investigating the reallocation of attention in a similar situation, Bristow et al. (2007) were able to identify a corresponding interaction effect. BOLD-responses in the parieto-frontal attentional network indicated a stronger reallocation of attention for the observation of gaze shifts toward empty space vs. an object when the observed face had previously looked at the participant in contrast to an averted gaze condition. The authors assumed that the enhanced (visual) saliency of eyes directed at the viewer might have increased the gaze cueing effect.

When participants had to rate whether or not the VC appeared to be interested in something (PRIV), only shift amplitude had a notable effect with larger gaze shifts eliciting higher approval rates. We assume that participants tended to perceive small gaze shifts as still directed toward them. Despite the human general acuteness in retracing gaze vectors and directions, they show a surprising tolerance when identifying gaze directed at them with deviations up to several degrees (Gibson and Pick, 1963; Jenkins et al., 2006; Mareschal et al., 2013). Interestingly, this tendency is even stronger for participants that had experienced social exclusion prior to the experiment (Lyyra et al., 2017). We, however, did neither induce or ask explicitly for the experience of social exclusion.

It makes sense that participants, when asked whether the other one was interested in something, assumed this something in the outside world and took more decisive gaze shifts as reflecting this interest. In general, humans, when observing another persons’ gaze, express some flexibility not only with regard to gaze directed at them, but also when it is directed at objects. We perceive a person as looking directly toward an object even in case of an actual divergence between gaze vector and object (Lobmaier et al., 2006). Unfortunately, research on the effect of the target position and shift amplitude in gaze cueing is still sparse. To the best of our knowledge, only one study investigated the gaze cueing effect as a function of the cued position, reporting higher effects for more distant positions (Qian et al., 2013). Our data now suggest, that when gaze shifts were more pronounced, participants more strongly imagined the existence of objects in their shared environment, even though not visible to them. However, due to the still insufficient knowledge about the underlying mechanisms this notion remains speculative.

It is interesting that the initial gaze does not influence the judgment. Even when initially eye contact was established, this did not impede the impression of privately motivated behavior so that the interpretation of the same behavior either as communicative or as private crucially depends on the instruction or the “mindset.” Obviously, private and communicative intentions are not mutually exclusive, a person can be interested in something and therefore try to show it to others. However, at least in this highly reductionistic quasi-“social” context, participants did not or were not able to distinguish between those two situations.

Taken together, results corroborate that the combination of mere eye contact and lateral gaze shift together can already signal communicative intentions in a very robust way and can serve as powerful ostensive cue. However, data suggest that eye contact itself and even in combination with the subsequent gaze shift are not sufficient to biuniquely discern intentions from social gaze. The impression of communicative intentions was most prevalent in, but not limited to, the most profiled triadic situations, defined by initial eye contact and large gaze shift amplitudes. This is in line with results showing that ostensive gaze cues do not necessarily seem to be a prerequisite for gaze following in infants (Szufnarowska et al., 2014; Gredebäck et al., 2018). Conversely, eye contact did not inhibit the impression of private intentions. With regard to the differentiation between communicative and private intentions, this means that eye contact neither seems to constitute a highly predictive nor selective signal. Thus, the question remains, which other signals or processes might be used discern intentions from gaze.

Here, the highly reductionist approach of this study clearly reaches its limits. While it was warranted for elucidating the relationship between the most basic aspects of ostensive gaze behavior, its limitations have to be considered as well. First: Non-verbal communication in general was already pointed out to have a high procedural and dimensional complexity meaning that individual non-verbal cues are not isolated units but always part of a stream of cues from different non-verbal channels (Vogeley and Bente, 2010). Regarding the investigation of gaze behavior it is thus advisable not to limit the analysis to short chunks of gaze communication and potentially to include other non-verbal channels as well (Jording et al., 2018). Second: The context or environment has to be taken into account when investigating gaze processing (Hamilton, 2016). Adding and systematically varying objects to the setup as a focus point for the ostensive gaze cues would thus constitute another interesting variation of this study. Third: Closely linked to environmental aspects are factors regarding our knowledge about the other person. Although gaze cueing and gaze following can happen automatically, it is also influenced by our perception and beliefs about the other person as well as our relationship toward this person (Gobel et al., 2017). Thus, systematically manipulating the participants believes about of the observed agent (e.g., personality or preferences) might influence their interpretation of the observed gaze behavior.

## Conclusion

In conclusion, although the two studies on gaze behavior presented here are highly minimalistic, they nevertheless substantially deepen our understanding of the powerful potential of social gaze in initiating interactions, referencing and displaying attention and thus allow a glimpse through the “window into social cognition” that social gaze can provide (Shepherd, 2010). Eye contact has again been proven to be a powerful tool in imparting communicative intents and fostering the impression that someone else is actively trying to show us something. However, it also becomes evident that eye contact itself is obviously not sufficient to discern intentions from social gaze biuniquely. Humans most likely make use of additional, e.g., temporal characteristics of gaze or they take other non-verbal or verbal signals into account; further investigations on this topic are therefore warranted. In practice, this study can inform us about the fundamental processes that underlie the perception and potentially production of gaze behavior and their functional roles in communication. Technically, these insights may help develop applications in the field of interaction and communication sciences by making use of anthropomorphic virtual agents and humanoids (Pfeiffer et al., 2013). In order for cognitive robots to become accepted as interaction partners by humans they have to share the human ability to generate and interpret informative gaze behavior as a two-way communicative act (Pönkänen et al., 2011; Gobel et al., 2015; Jording et al., 2018). A more thorough understanding of how humans convey and ascribe intentions as supplied here is therefore essential. In the long-run this approach might then also foster the development of more sophisticated agent-based diagnostic and therapeutic instruments for communication disorders like autism spectrum disorders (Georgescu et al., 2014).

## Data Availability Statement

Data will be made available in a public repository upon publication and till then can be accessed via https://osf.io/avu5w/?view_only=bd507466e05544589eac294c33253e8c.

## Ethics Statement

This study followed the WMA Declaration of Helsinki (Ethical Principles for Medical Research Involving Human Subjects) and was presented to and approved by the Ethics Committee of the Medical Faculty of the University Hospital Cologne, Germany.

## Author Contributions

All authors substantially contributed to the conception of the work. DE programed the code for data collection in the online and in the lab study. HE recruited participants and conducted the lab experiment. MJ and DE conducted the statistical analyses. MJ drafted the manuscript. HE, DE, GB, and KV revised the manuscript critically.

## Conflict of Interest

The authors declare that the research was conducted in the absence of any commercial or financial relationships that could be construed as a potential conflict of interest.

## Supplementary Material

FIGURE S1 Illustration of the female avatar’s eyes and the measurement of the iris’ position for gaze angle calculation. Middle column: Eye section from the female avatar stimuli for the initial gaze position (top four) and the position after the gaze shift (bottom four). Red circles with a centered cross mark the position of the iris as measured for the calculation of the gaze angle. Right column: lateral and horizontal deviations of the gaze angle from direct gaze. Note that depicted here are only gaze shifts to the left side; for shifts to the right side avatar stimuli were mirrored.

FIGURE S2 Illustration of the male avatar’s eyes and the measurement of the iris’ position for gaze angle calculation. Middle column: Eye section from the female avatar stimuli for the initial gaze position (top four) and the position after the gaze shift (bottom four). Red circles with a centered cross mark the position of the iris as measured for the calculation of the gaze angle. Right column: lateral and horizontal deviations of the gaze angle from direct gaze. Note that depicted here are only gaze shifts to the left side; for shifts to the right side avatar stimuli were mirrored.

TABLE S1 Coefficients sampled from the approximate posterior distribution in Study 1 for the influence of the COM condition, initial position, shift amplitude, and their respective interactions. The COM coefficient describes the effect of the COM condition in contrast to the LOOK condition; init. pos. depicts the stepwise effect of increasing aversion from direct gaze in the initial position (farther from direct gaze); gaze shift depicts the stepwise effect of increasing the shift amplitude. Reported are estimates (Estimate) and estimated errors (Est.Error) for the coefficients, the lower (l-95% CI) and the upper (u-95% CI) border of the 95% posterior compatibility intervals, the effective sample size (Eff.Sample) and the potential scale reduction factor $\hat{R}$ or Gelman-Rubin statistic ($\hat{R}$).

TABLE S2 Coefficients sampled from the approximate posterior distribution in study 2 for the influence of the COM condition, initial position, shift amplitude, and their respective interactions. The COM coefficient describes the effect of the COM condition in contrast to the PRIV condition; init. pos. depicts the stepwise effect of increasing aversion from direct gaze in the initial position (farther from direct gaze); gaze shift depicts the stepwise effect of increasing the shift amplitude. Reported are estimates (Estimate) and estimated errors (Est.Error) for the coefficients, the lower (l-95% CI) and the upper (u-95% CI) border of the 95% posterior compatibility intervals, the effective sample size (Eff.Sample) and the potential scale reduction factor $\hat{R}$ or Gelman-Rubin statistic ($\hat{R}$).

VIDEO S1 Example Study 1_init.1_shift.4.

VIDEO S2 Example Study 1_init.2_shift.3.

VIDEO S3 Example Study 1_init.3_shift.2.

VIDEO S4 Example Study 1_init.4_shift.1.

VIDEO S5 Example Study 2_init.1_shift.4.

VIDEO S6 Example Study 2_init.2_shift.3.

VIDEO S7 Example Study 2_init.3_shift.2.

VIDEO S8 Example Study 2_init.4_shift.1.
