# Supplementary material for: Distinguishing Social From Private Intentions Through the Passive Observation of Gaze Cues
Source: Front Hum Neurosci. 2019 Dec 17;13:442. doi: 10.3389/fnhum.2019.00442 (PMC6928136; doi:10.3389/fnhum.2019.00442)
Supplement: FIGURE S1 — Illustration of the female avatar’s eyes and the measurement of the iris’ position for gaze angle calculation. Middle column: Eye section from the female avatar stimuli for the initial gaze position (top four) and the position after the gaze shift (bottom four). Red circles with a centered cross mark the position of the iris as measured for the calculation of the gaze angle. Right column: lateral and horizontal deviations of the gaze angle from direct gaze. Note that depicted here are only gaze shifts to the left side; for shifts to the right side avatar stimuli were mirrored. [file Data_Sheet_1.PDF]

Supplementary Material

Content

Figure S-1. Illustration of the female avatar‘s eyes and the measurement of the iris‘ position for gaze angle calculation. 2

Figure S-2. Illustration of the male avatar‘s eyes and the measurement of the iris‘ position for gaze angle calculation. 3

Table S-1. Coefficients sampled from the approximate posterior distribution in study 1 for the influence of the COM condition, initial position, shift amplitude, and their respective interactions..... 4

Table S-2. Coefficients sampled from the approximate posterior distribution in study 2 for the influence of the COM condition, initial position, shift amplitude, and their respective interactions..... 5

Female Avatar

Initial Gaze

Female Avatar

Gaze shift

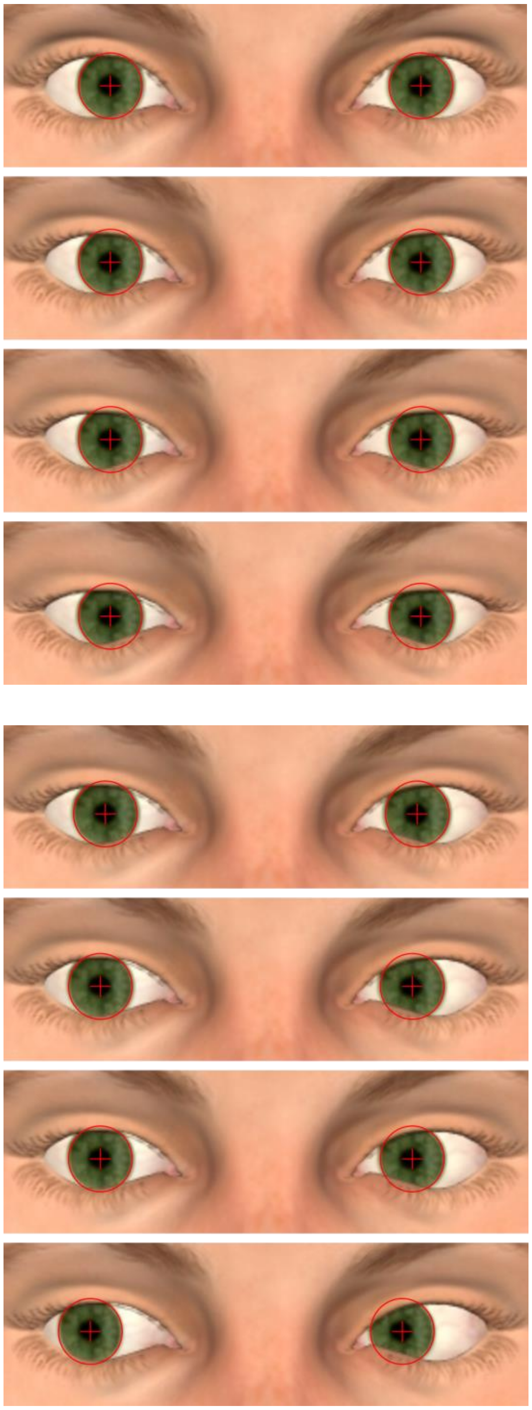

Initial position 1 (direct gaze)

Lateral deviation: 0°
Horizontal deviation: 0°

Initial position 2

Lateral deviation: 0°
Horizontal deviation: 3.64°

Initial position 3

Lateral deviation: 0°
Horizontal deviation: 8.23°

Initial position 4

Lateral deviation: 0°
Horizontal deviation: 12.13°

Shift position 1

Lateral deviation: 6.49°
Horizontal deviation: 4.59°

Shift position 2

Lateral deviation: 6.49°
Horizontal deviation: 9.26°

Shift position 3

Lateral deviation: 6.49°
Horizontal deviation: 14.21°

Shift position 4

Lateral deviation: 6.49°
Horizontal deviation: 18.10°

Figure S-1. Illustration of the female avatar‘s eyes and the measurement of the iris‘ position for gaze angle calculation. Middle column: Eye section from the female avatar stimuli for the initial gaze position (top four) and the position after the gaze shift (bottom four). Red circles with a centered cross mark the position of the iris as measured for the calculation of the gaze angle. Right column: lateral and horizontal deviations of the gaze angle from direct gaze. Note that depicted here are only gaze shifts to the left side; for shifts to the right side avatar stimuli were mirrored.

Male Avatar

Initial Gaze

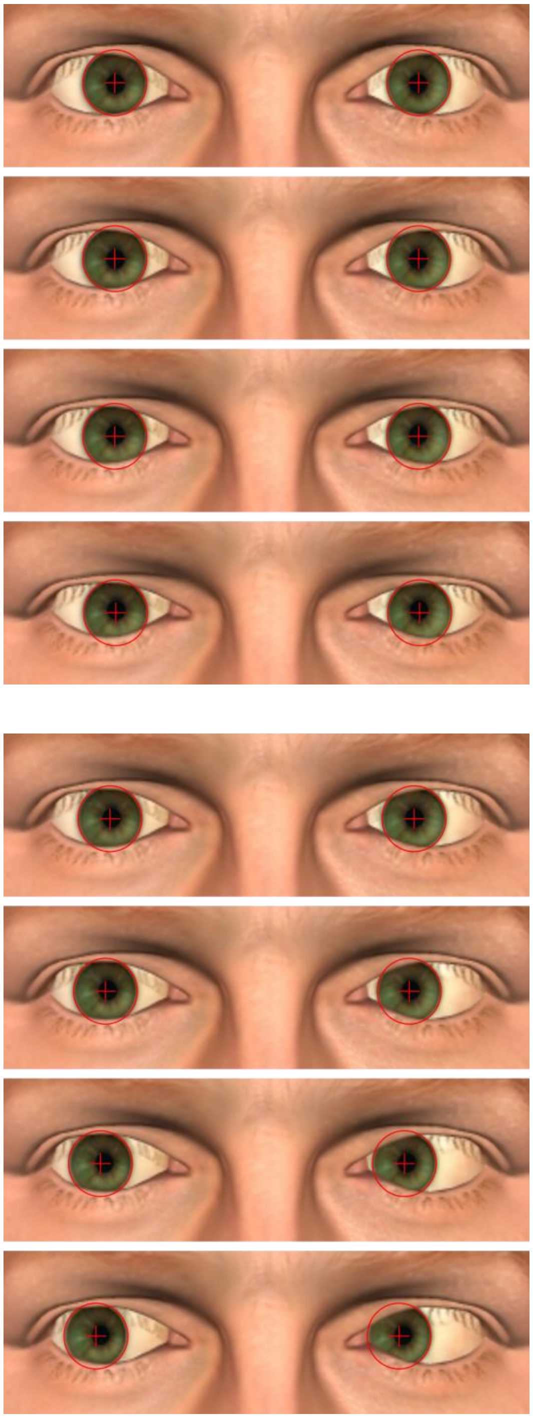

Initial position 1 (direct gaze)

Lateral deviation: 0°
Horizontal deviation: 0°

Initial position 2

Lateral deviation: 0°
Horizontal deviation: 3.31°

Initial position 3

Lateral deviation: 0°
Horizontal deviation: 8.31°

Initial position 4

Lateral deviation: 0°
Horizontal deviation: 12.04°

Male Avatar

Gaze shift

Shift position 1

Lateral deviation: 6.21°
Horizontal deviation: 4.47°

Shift position 2

Lateral deviation: 6.21°
Horizontal deviation: 9.03°

Shift position 3

Lateral deviation: 6.21°
Horizontal deviation: 13.47°

Shift position 4

Lateral deviation: 6.21°
Horizontal deviation: 18.20°

Figure S-2. Illustration of the male avatar‘s eyes and the measurement of the iris‘ position for gaze angle calculation. Middle column: Eye section from the female avatar stimuli for the initial gaze position (top four) and the position after the gaze shift (bottom four). Red circles with a centered cross mark the position of the iris as measured for the calculation of the gaze angle. Right column: lateral and horizontal deviations of the gaze angle from direct gaze. Note that depicted here are only gaze shifts to the left side; for shifts to the right side avatar stimuli were mirrored.

	Estimate	Est.Error	l-95% CI	u-95% CI	Eff.Sample	$\hat{R}$
**Intercept**	0.3473	0.0886	0.1669	0.5250	1592.1690	1.0032
**COM**	-0.4370	0.1229	-0.6778	-0.1946	1445.5120	1.0021
**init. pos.**	-1.9616	0.0651	-2.0889	-1.8359	3387.4570	0.9992
**gaze shift**	-0.0933	0.0501	-0.1887	0.0068	3327.5410	1.0005
**COM × init. pos.**	1.3199	0.0768	1.1675	1.4690	3635.8090	0.9993
**COM × gaze shift**	0.7367	0.0656	0.6105	0.8683	3265.7210	1.0008
**init. pos. × gaze shift**	-0.1615	0.0610	-0.2812	-0.0428	3764.6170	0.9997
**COM × init. pos. × gaze shift**	0.1910	0.0754	0.0416	0.3339	3719.7810	1.0000

Table S-1. Coefficients sampled from the approximate posterior distribution in study 1 for the influence of the COM condition, initial position, shift amplitude, and their respective interactions. The COM coefficient describes the effect of the COM condition in contrast to the LOOK condition; init. pos. depicts the stepwise effect of increasing aversion from direct gaze in the initial position (farther from direct gaze); gaze shift depicts the stepwise effect of increasing the shift amplitude. Reported are estimates (Estimate) and estimated errors (Est.Error) for the coefficients, the lower (l-95% CI) and the upper (u-95% CI) border of the 95% posterior compatibility intervals, the effective sample size (Eff.Sample) and the potential scale reduction factor $\hat{R}$ or Gelman-Rubin statistic ($\hat{R}$).

	Estimate	Est.Error	l-95% CI	u-95% CI	Eff.Sample	$\hat{R}$
**Intercept**	0.0785	0.1398	-0.1978	0.3430	416.4663	1.0047
**COM**	-0.2136	0.0690	-0.3485	-0.0758	5803.8174	0.9995
**init. pos.**	-0.0162	0.0472	-0.1076	0.0751	3570.5366	1.0007
**gaze shift**	0.4761	0.0473	0.3841	0.5682	3484.6917	0.9997
**COM × init. pos.**	-0.5381	0.0699	-0.6743	-0.3994	3422.5331	0.9998
**COM × gaze shift**	0.3174	0.0701	0.1793	0.4536	3251.7205	1.0006
**init. pos. × gaze shift**	-0.0294	0.0470	-0.1201	0.0621	3364.8641	1.0003
**COM × init. pos. × gaze shift**	-0.0157	0.0701	-0.1521	0.1198	2789.1807	1.0009

Table S-2. Coefficients sampled from the approximate posterior distribution in study 2 for the influence of the COM condition, initial position, shift amplitude, and their respective interactions. The COM coefficient describes the effect of the COM condition in contrast to the PRIV condition; init. pos. depicts the stepwise effect of increasing aversion from direct gaze in the initial position (farther from direct gaze); gaze shift depicts the stepwise effect of increasing the shift amplitude. Reported are estimates (Estimate) and estimated errors (Est.Error) for the coefficients, the lower (l-95% CI) and the upper (u-95% CI) border of the 95% posterior compatibility intervals, the effective sample size (Eff.Sample) and the potential scale reduction factor $\hat{R}$ or Gelman-Rubin statistic ($\hat{R}$).
